# Incidence of symptomatic COVID-19 in close contacts of patients after discharge from hospital

**DOI:** 10.1186/s12879-022-07300-x

**Published:** 2022-03-26

**Authors:** Ayat Ahmadi, Amirhossein Poopak, Sina Nazemi, Negin Mohammadi, Bita Eslami, Monireh Sadat Seyyedsalehi, Leila Doshmangir, Seyyed Farshad Allameh, Kazem Zendehdel

**Affiliations:** 1 Knowledge Utilization Research Center, Tehran University of Medical Sciences, Tehran, Iran; 2 Cancer Research Center, Cancer Institute, Tehran University of Medical Sciences, Tehran, Iran; 3 Radiation Oncology Research Center, Cancer Institute, Tehran University of Medical Sciences, Tehran, Iran; 4 Breast Disease Research Center, Cancer Institute, Tehran University of Medical Sciences, Tehran, Iran; 5 Social Determinants of Health Research Center, Tabriz University of Medical Sciences, Tabriz, Iran; 6 Department of Health Policy & Management, Tabriz Health Services Management Research Center, Tabriz University of Medical Sciences, Tabriz, Iran; 7 Department of Gastroenterology, Tehran University of Medical Sciences, Tehran, Iran

**Keywords:** COVID-19, Patients discharge, Close-contact transmission, Comorbidity

## Abstract

**Background:**

There is a little evidence about the infectiousness of recovered COVID-19 patients. Considering that the circumstance of the isolation of the COVID-19 patients after-discharge is not always optimal, it is not very unlikely that viral transmission still occurs after hospital discharge. This study aims to investigate the incidence of symptomatic COVID-19 in close contacts of recovered patients after discharge from hospital.

**Methods:**

Four hundred fifty discharged COVID-19 patients discharged from the largest public treatment center in Tehran, capital city of Iran, were followed up. Demographic and clinical data of participants were collected from medical records. Follow-up data were acquired via telephone call interviews with patients or their main caregivers at home.

**Results:**

The study’s response rate was 93.77% (422 participated in the study). 60.90% patients were male and 39.10% were female (sex ratio = 1.55 male). The most prevalent comorbidities in these patients were hypertension (29.68%) and diabetes (24.80%). The mean of home isolation after discharge was 25.85. Forty-one (9.71%) patients had at least one new case in their close contacts, up to 3 weeks after they were discharged. There was a significant association between having at least a comorbidity with the odds of getting infected in close contacts [OR (CI) 2.22 (1.05–4.68)]. Density of inhabitant per room in a house’ and the quality of isolation had significant associations with observing new cases in the patients’ close contacts [high to moderate; OR (CI) 2.44 (1.06–5.61], [bad to good; OR (CI) 2.31 (1.17–4.59)], respectively.

**Conclusion:**

After hospital discharge, COVID-19 transmission can still occur, when a large number of people lives together in a single house. Another explanation can be that the less precaution measures are taken by recovered patients’ cohabitants. Such conditions are also likely to happen when the recovered patient has other chronic diseases and requires additional care.

## Background

The coronavirus disease (COVID-19) caused by the novel coronavirus (SARS-CoV-2) represents a significant global medical issue, with a growing number of cumulative confirmed cases [1]. COVID-19 is characterized by a high transmissibility before and immediately after symptom onset that changed it into an acute problem in the world [2]. Until 20 September, 2021, the confirmed COVID-19, deaths and recovered cases have been reported 275,036,329; 5,371,123 and 246,819,001 respectively [3] all over the world. Countries and particularly health systems selected various policies and clinical measures to control COVID-19 transmission [4]. Better understanding of the transmission dynamics of the virus is important for the development and evaluation of effective control measures [2]. The primary criteria to allow discharge of symptomatic Covid-19 patients from isolation was based on RT-PCR test result and the estimation of the duration of viral shedding after recovery. This recommendation stemmed from former knowledge about other coronaviruses [5]. It would require patients to be tested twice at least 24 h apart [5]. However, in updated guidelines, the balance between risks and benefits of patients’ health has become the forefront of recommendations for the time of discharge, so then, the time point when symptomatic patients are no longer considered infectious, has been defined according to the time when disease symptoms start and disappear. This recommendation became paramount since the previous one came out infeasible in many settings [6]. It also aims to avoid unnecessary isolation time periods and extra clinical tests [7]. These criteria are recommended according to studies which have shown that the viral load of recovered patients declines quickly [8].

However, the criteria for hospital discharge vary widely based on the capacity of services in hospitals and definitions of clinical recovery [9]. Consequently, criteria for discharge patients from hospital are not the same as the criteria for discharge patients from isolation [10]. About half of clinically recovered COVID-19 patients have a positive test result at the time of recovery, and some have the RT-PCR positive results prolonged up to 14 days after recovery [11]. Expectedly, there is potential for the viral transmission after hospital discharge, especially when the circumstance of the isolation is not optimal [12]. Cheng et al. [2] showed that discharge from hospital should not be considered the endpoint of monitoring and precautionary measures and the potency of transmission of virus to others should not be underestimated [1].

Providing further evidence to improve isolation criteria for recovered patients and establish the criteria for the post-discharge isolation condition will help to fade the possible risks of viral transmission away. Finding new cases among close contacts of discharged patients, during a few weeks after discharge, will show the possibility of viral transmission and the condition in which the transmission is more probable to occur. In doing so, we studied the rate of the incidence of new cases of COVID-19 who had close contacts with recovered and discharged COVID-19 patients and its potential associated factors.

## Methods

We recorded 450 hospitalized patients in Imam Khomeini hospital complex, the largest educational referral center in Iran with three hospitals and more than 1200 beds. The considered patients were those who were hospitalized following the covid-19 clinically compatible illness, had a positive RT-PCR test result, and were discharged from the hospital because their main symptoms went away and their recovery was clinically confirmed. A data collection tool was prepared according to study objectives. The tool had two main parts: the first part included demographic and clinical data, which were retrieved from hospital records, and the second part included follow-up data which were collected through telephone call interviews with discharged patients or their main caregivers. The follow-up data included patients’ symptoms after discharge, their current health status, and any confirmed new cases in their close contacts. Telephone calls were made by three interviewers and were repeated up to three times in different hours and days to increase the response rate. Patients were registered as non-response, if they did not answer all the calls. Furthermore, patients that answered telephone calls but were not interested in participation in the study were registered as non-response as well.

### Statistical analysis

Patients’ characteristics were described by the mean and standard deviation or frequency and percent. The incidence of new cases of covid-19 was defined as hospitalized or having positive test results for covid-19 in close contacts. Those patients who stated that they had at least one new case of covid-19 in their close contacts within 3 weeks, after their discharge time (considered cases) were compared to participants who stated no new case of covid-19 in their close contacts (considered controls). Cases and controls were described and were compared regarding potential effective factors. House density was defined as the number of patients’ cohabitants in a same house divided by the number of rooms in the house. Educational level was defined in four levels including illiterate or elementary, lower intermediate, upper intermediate and academic. The quality of isolation was defined as the staying in an independent room in the home for at least 10 days with separated meals and the patient’s own point of view about his quality of isolation after discharge. Comorbidity variable was defined as *“having at least a comorbidity*”. The associations of the independent variables with the outcome were estimated using univariate and multivariable logistic regression. In the multivariable model we applied a backward stepwise selection (p < 0.1 for entry and p ≥ 0.2 for removal) [13]. We used the relevant command in stata as below:

xi: sw, pr(0.1) pe(0.05): logistic incidence (i.r_age) sex education comorbidity hospital_dura symptoms visit_health_service return_job days_not_goout (i.density) isolation_quality

All analysis was performed in stata14 (Stata Statistical Software: Release 14. College Station, TX: StataCorp LLC).

## Results

Among 450 COVID19 patients, 422 answered the call and agreed to be interviewed (response rate: 93.77%). Demographic and potential predictors of infectivity are shown in Table 1. Out of 262 patients with recorded comorbidities, the most prevalent comorbidities were hypertension (42.36%), followed by diabetes (35.49%), hyperlipidemia (29.00%) and history of cardiovascular disease (18.70%). The mean of staying at home (home isolation) after discharge was 25.85 days (Table 1).

**Table 1:** Demographic and co-morbidities of COVID-19 patients participated in this study (n = 319)

Study’s variables	Number (%)
Sex	
Male	257 (60.90)
Female	165 (39.10)
Education level	
Illiterate or elementary	98 (23.31)
Under intermediate	83 (19.84)
Upper intermediate	140 (32.71)
Academic	101 (23.96)
Age (years)	
< 40	94 (22.38)
40–50	73 (17.38)
50–60	100 (23.81)
60–70	89 (21.91)
70<	64 (15.24)
Co-morbidities (n = 262, 62.08)	
HTN	111 (42.36)
Diabetes	93 (35.49)
Hyperlipidemia	76 (29.00)
Cardiovascular	49 (18.70)
Cancer	34 (12.97)
Renal	17 (6.48)
Asthma	16 (6.10)
Intestinal	14 (5.34)
Liver	11 (4.19)
COPD	6 (2.29)
CVA	3 (1.32)
Hospital duration (days)	
< 3	215 (50.94)
3–7	141 (33.41)
7–10	37 (8.76)
10<	28 (6.63)
Having symptoms after discharge	
Yes	252 (59.71)
No	170 (40.28)
Days did not go out after discharge (weeks)	
< 2	111 (26.30)
2–3	142 (33.65)
3–5	116 (27.49)
5<	53 (12.56)
Visited after discharge	
Yes	220 (52.13)
No	147 (34.83)
Not remembered	55 (13.03)
Return to job	
Yes	85 (27.24)
No	227 (72.76)
Quality of isolation	
Good	255 (60.42)
Bad	79 (18.72)
Not answered	88 (20.8)
House density	
Low	138 (32.70)
Moderate	218 (51.66)
High	66 (15.64)

Within 3 weeks since the discharge, 41 (9.71%) participants had at least one new case in their close contacts. Among others, 72.74% stated that there was no new case in their close contacts. The rest of the patients (17.53%), did not know whether there was any new case in their closes or not. Of those who had new cases in their close contacts, 73.17% stated that there was one new case, 19.51% stated that there were two new cases, and 7.31% stated that there were three new cases in their close contacts. Piling up, there were 55 symptomatic new cases which can potentially have got the infection from 41 discharged patients within 3 weeks of their time of discharge. The majority of new cases were male (59.25%) and the mean of their age was 39.35.

Those patients who had at least one new case of covid-19 in their close contacts (n = 41) were compared to participants who stated that there was no new case in their contacts (n = 307) (control to case ratio 7.48:1).

The density of inhabitant per houses rooms, [high density to moderate density; OR (CI) 2.44 (1.06–5.61)], the quality of isolation [bad condition to good condition; OR (CI) 2.31 (1.17–4.59)], and having at least a comorbidity [Yes to No; OR (CI) 2.22 (1.05–4.68)] had significant associations with potential transmission in close contacts. These three variables were the only variables that remained in the final model of our backward stepwise modeling (Table 2). The final regression model was:

**Table 2:** Crude and age–sex–education-adjusted associations for potential determinant factors and the incidence of the symptomatic COVID-19 in close contacts of COVID-19 recovered patients, within 3 weeks after discharge

Potential determinant factors of being a source of infection	New case in closed contacts; n (%)		Crude OR (95% CI)	Adjusted odds ratio (CI)
	Yes; 41 (11.78)	No; 307 (88.22)		
Age (year), mean (± sd)	54.19 (15.90)	53.72 (15.98)	1.00 (0.98–1.02)	–
Sex				
Female	19 (46.34)	107 (34.85)	1	–
Male	22 (53.66%)	200 (65.15)	0.62 (0.32–1.19)	–
Level of education				
Low	23 (56.10)	151 (86.78)	1	–
High	18 (43.90)	138 (86.78)	1.16 (0.60–2.25)	–
Comorbidity				
No	10 (2.87)	128 (36.78)	1	1
Yes	31 (8.91)	179 (51.44)	2.22 (1.05–4.68)*	2.48 (1.13–0.45)*
Hospital duration				
Less than 3 days	21 (51.22)	150 (49.02)	1	–
More than 3 days	20 (48.78)	156 (50.98)	0.91 (0.47–1.75)	–
Having symptoms after discharge				
No	15 (36.59)	107 (34.85)	1	–
Yes	26 (63.41)	200 (65.15)	0.92 (0.47–1.82)	–
Visit health centers after discharge				
No	29 (72.50)	177 (59.60)	1	–
Yes	11 (27.50)	120 (40.40)	0.55 (0.26–1.16)	–
Return to job				
No	26 (74.29)	194 (72.12)	1	–
Yes	9 (25.71)	75 (27.88)	0.89 (0.40–1.99)	–
Days not to go out				
Less than 3 weeks	25 (60.98)	165 (53.75)	1	–
More than 3 weeks	16 (39.02)	142 (46.25)	0.74 (0.38–1.44)	–
Home density				
Moderate	14 (34.15)	151 (49.19)	1	1
Low	15 (36.59)	103 (33.55)	1.57 (0.72–3.39)	1.58 (0.71–3.49)*
High	12 (29.27)	53 (17.26)	2.44 (1.06–5.61)*	2.80 (1.18–6.62)
Quality of isolation				
High	24 (58.54)	62 (23.40)	1	1
Low	17 (41.46)	203 (76.60)	2.31 (1.17–4.59)*	2.20 (1.09–4.45)*

– Dropped from the final model

^*^Significant at level of 0.05

xi: sw, pr(0.1) pe(0.05): logistic incidence comorbidity (i.density) isolation_quality.

The estimated parameters in the model were as below (in logit term):

logit (odds of the outcome) = − 3.05 + 0.9 (Comorbidity) + [1.03 (density: high/moderate) + 0.46 (density: low/moderate)] + 0.79 (Quality of isolation).

## Discussion

Our follow up study on discharged COVID-19 patients showed that there is a potential chance of viral transmission to patients’ close contacts within 3 weeks of discharge. Our findings also implied that the probability of the incidence of new infected cases will go up when the discharged patient has chronic comorbidities and when the quality of isolation is low. These findings show that although the risk of infectivity in recovered patients can fairly be considered low, having effective contact with such patients might lead to the transmission of the infection. Since the data about discharged COVID-19 patients and their closed contacts are not widely available [1, 14, 15], these results can be considered meaningful for the providing of sound recommendations for discharged patients.

We found that, adjusted for other variables, the risk of the incidence of symptomatic COVID-19 among close contacts of recovered patients with at least a comorbidity, was 2.22 times higher compared to those patients who had no comorbidity. Although this association can be considered relatively strong, the lower bound of the odds ratio approximates to 1 (the null hypothesis) and therefore any possible interpretation should remain conservative. Previous studies implied that comorbidities will increase the chance of infection [16] and COVID-19 patients with other comorbidities are more likely to develop more severe courses of the disease [17], however, the infectivity of COVID-19 patients with different types of comorbidities has not been extensively explored [18, 19]. In fact, the vast majority of evidence which investigated the determinants of COVID-19’ infectivity has focused on the virus-related factors, such as viral shedding and the presence of replication-competent viruses, as influential factors for the infection transmission [20]. The observed relationship between comorbidity and the infectivity of COVID-19 patients in this study, can be explained by the level of in-person care that patients needed. According to our result, the higher level of care patients need, probably, the more physical proximity and more interaction between patients and their close contacts will occur. This physical proximity seems to be more determinant for infectivity and viral transmission [19, 21].

The result of this study also showed that having new cases in close contacts of patients who lived within crowded households was more than threefold higher than patients with a moderate living density. This finding is comparable with other studies that showed the household secondary attack rate of COVID-19 in high living density households is about 3 times more [19, 22]. Likewise, the risk of infectivity for discharged patients who lived in the low quality of isolation after discharge was more than twice as much as patients who lived in higher quality of isolation. These findings stress on the specific behavioral and residential condition that COVID-19 patients experience after discharge from hospitals. This condition may become more important when patients’ close contacts intend to spend more time with discharged patients, for example when discharged patients need some in-person care by his or her caregivers. The mentioned finding is compatible with other evidence which imply that physical distancing and verbal interactions are the determinant factors for viral transmission [19, 23, 24]. It is also consistent with Luo [25] findings that pointed out the household transmission as the main route of the transmission of COVID-19. Not very surprisingly, these associations were independent of patients’ gender and age. It is worth noting that in this study, we explored the effect of the characteristics of the infector, such as age and sex, on the infectivity and transmission which, compare to infected patients, have been investigated less in other studies [18]. In our study, in spite of the lower rate of symptomatic COVID-19 among close contacts of male patients, we found no significant relationship for sex and age with the study outcome. These results are compatible with Hu and colleagues [19] which have shown that the age and sex of infectors are not in association with the infectiousness. In their study, the observed higher risk of infectivity in people aged 15–64 years disappeared in the multivariable analysis [19].

In this study the incidence of new cases in close contacts was measured via the patients’ statement. It is identifiable that patients’ statement is not the optimal measure for establishing the transmission link between new patients and the discharged patients. It means that the observed new cases in close contacts of discharged patients, might have got the infection from other sources. Having said that, there might be new cases around discharged patients that were missed falsely, either for being asymptomatic or simply missed by the interviewee. Nevertheless, the denominator for calculating the risk of observing symptomatic patients in close contacts of discharged patients was not established in this study, the average risk of infection in the society in the same period was too small to be considered influential. In addition, due to sample size limitation, we combined different chronic comorbidities in one variable so as to have enough sample size and avoid complexity in statistical models. Therefore, we could not apply all comorbidities in the models. It is also worth mentioning that criteria for the recovery from the disease are somehow changing time by time, depending on the availability of tests, accessibility of services and the rate of new cases who need to become hospitalized [9, 12]. As a consequence, a patient who was considered recovered and was discharged at a specific time might have not been considered so, if he had got the disease at a different time and different circumstances.

## Conclusion

Finding a fairly high number of new cases in close contacts of discharged patients shows that considering recovered patients thoroughly out of the risk of transmission might not be quite safe, especially for those contacts that are in touch with the patients in longer durations of time. Such conditions are more likely to happen when the recovered patients suffer from chronic comorbidities and need to be cared for by their close contacts or when patients live in higher household densities. We also found that discharged patients can become a source of infection for others when the quality of isolation is low and the strictness of precautions is not considered serious.

## Data Availability

All supportive data are available within the article and its additional files.
